# Freezing of gait in Chinese patients with multiple system atrophy: prevalence and risk factors

**DOI:** 10.3389/fnins.2023.1194904

**Published:** 2023-06-07

**Authors:** Yalan Chen, Hui Wang, Hongyan Huang, Yangmei Chen, Yanming Xu

**Affiliations:** ^1^Department of Neurology, The Second Affiliated Hospital of Chongqing Medical University, Chongqing, China; ^2^Department of Neurology, West China Hospital, Sichuan University, Chengdu, Sichuan, China

**Keywords:** multiple system atrophy, freezing of gait, prevalence, risk factor, motor symptoms

## Abstract

**Objective:**

Freezing of gait (FOG) is common in neurodegenerative forms of atypical parkinsonism, but few studies have examined FOG in multiple system atrophy (MSA). In this study, we examined the prevalence of freezing of gait and its relationship to clinical features in a large cohort of Chinese MSA patients.

**Methods:**

This exploratory study included 202 Chinese patients with probable MSA. FOG was defined as a score ≥ 1 on item 14 of the Unified Parkinson’s Disease Rating Scale. Patients with or without FOG were compared in terms of the Unified MSA Rating Scale (UMSARS) as well as cognitive and neuropsychiatric assessments.

**Results:**

The frequency of FOG was 48.0, 52.1, and 38.7% in MSA, MSA with predominant parkinsonism (MSA-P), and MSA with predominant cerebellar ataxia (MSA-C), respectively. FOG was associated with worse subscores on parts I, II and IV of the UMSARS as well as worse total UMSARS score; greater likelihood of speech difficulties, falls, gait impairment and balance disorder; more severe symptoms of anxiety and depression; and lower activities of daily living. The binary logistic regression model indicated that higher total UMSARS scores were associated with FOG in MSA, MSA-P, and MSA-C patients.

**Conclusion:**

Freezing of gait may be common among Chinese MSA patients, FOG may correlate with severe motor symptoms, anxiety, depression and activities of daily living. Total UMSARS score may be an independent risk factor for FOG.

## Introduction

Freezing of gait (FOG) is a disabling gait disorder defined as “brief, episodic absence or marked reduction of forward progression of the feet despite the intention to walk” (Nutt et al., 2011). Patients with FOG describe a feeling that their feet are “glued” or “magnetically stuck” to the floor. FOG has traditionally been associated with Parkinson’s disease (PD), yet it is more common in atypical forms of parkinsonism, such as progressive supranuclear palsy and multiple system atrophy (MSA) (Factor, 2008; Rezvanian et al., 2020; Cosentino et al., 2023).

However, limited studies have reported the prevalence of FOG in patients with MSA. A retrospective analysis of 28 patients with MSA revealed that 75% of the patients had complained of FOG (Gurevich and Giladi, 2003), whereas another cross-sectional study, which enrolled 91 patients with MSA, found that 65.9% of patients had FOG (Yang et al., 2022). Additionally, patients who experience FOG might encounter worse motor impairment and poorer quality of life. Besides, the sample size was small and the samples were heterogeneous in the above studies. Studies with a larger sample size are required to further address the impact of FOG in patients with MSA. Therefore, the present study examined the prevalence of FOG in a relatively large cohort of Chinese MSA patients, and it explored what clinicodemographic factors may be associated with FOG.

## Materials and methods

### Study population

This study retrospectively examined patients who met the clinical diagnostic criteria (Gilman et al., 2008) for probable MSA and were admitted to West China Hospital of Sichuan University and The Second Affiliated Hospital of Chongqing Medical University from February 2016 to June 2021. Patients were excluded if they had other neurodegenerative disorders, were unable to communicate, or refused to participate in the study. This study was approved by the Ethics Committee of West China Hospital of Sichuan University and informed consent was obtained from all patients prior to enrollment.

### FOG evaluation

Freezing of gait was evaluated using item 14 (“Freezing”) of the Unified Parkinson’s Disease Rating Scale (UPDRS) (Martinez-Martin et al., 1994). Scores on this item can range from 0 to 4, with a higher score indicating more severe freezing. Patients were asked if they ever felt that their feet were “glued” to the floor when they tried to walk, or if they ever had problems when starting to walk or when turning. Responses were scored as follows: 0, “no freezing”; 1, “rare freezing when walking, may hesitate at the start”; 2, “occasional freezing when walking”; 3, “frequent freezing, occasionally falling due to freezing”; or 4, “frequent falls due to freezing.” Patients scoring at least 1 were diagnosed with FOG.

### Other assessments

Each patient was examined by a neurologist in a standardized structured interview. Data were collected on sex, age, age at MSA onset, MSA duration, smoking and drinking habits, urinary incontinence, inability to focus, concurrent diseases (Hypertension and Diabetes mellitus), and current medications. History of smoking behavior was indicated by at least one cigarette per day for 6 months or longer. History of drinking was defined as an average alcoholic drink ≥50 mL at least once per week lasting more than half a year. Hypertension was defined as a systolic BP ≥ 140 mmHg or diastolic BP ≥ 90 mmHg in the seated position, self-reported use of antihypertensive medications, or lifetime diagnosis of hypertension. Subjects were classified as having MSA-P if they exhibited parkinsonism signs without cerebellar features, or as having MSA-C if they displayed predominantly cerebellar signs with minimal or no parkinsonism, and if the cerebellar signs preceded any parkinsonism by at least 1 year (Low et al., 2015).

Patients were scored on parts I (historical review), II (motor examination scale), and IV (global disability scale) of the Unified MSA Rating Scale (UMSARS) (Wenning et al., 2004). Higher subscores for these parts indicated a worse disease state, as did a higher total UMSARS score, defined as the sum of subscores on parts I and II. Patients were also assessed according to whether they reported speech difficulties (UMSARS part I item 1 ≥ 2), falls (UMSARS part I item 8 ≥ 2), and gait impairment (UMSARS part II item 14 ≥ 2). A total score for balance was obtained by summing the subscores for item 8 (falling) on UMSARS part I and for item 13 (postural instability) on UMSARS part II.

Patients were also assessed using the Hamilton Depression Rating Scale (HAMD) and Hamilton Anxiety Rating Scale (HAMA). High scores on either instrument suggested greater levels of depression or anxiety symptoms, respectively. Sleep quality was assessed using the Pittsburgh Sleep Quality Index (PSQI) (Buysse et al., 1989), on which a higher score reflected lower sleep quality. Cognitive assessment was performed using the Mini-Mental State Examination (MMSE) (Folstein et al., 1975). Activities of daily living (ADL), an index of the ability to take care of oneself, were evaluated using part II of the UPDRS (Martinez-Martin et al., 1994).

### Statistical analysis

Continuous variables were reported as mean and standard deviation, and categorical variables as counts and percentages. Student’s *t*-test was used to compare continuous variables between patients with and without FOG in the MSA group, MSA-P subgroup, and MSA-C subgroup. The chi-squared or Fisher’s exact tests was used to compare the categorical variables between different groups. Spearman’s correlation analysis was performed to identify potential relationships of FOG frequency and severity with motor and non-motor symptoms. Multivariate binary logistic regression models were used to determine the factors related to FOG. Variables associated with FOG at *p*-values < 0.05 or possibly associated with FOG based on clinical judgment in the univariate analysis were included in the multivariate binary logistic regression model. Results were expressed as odds ratios (ORs) and 95% confidence intervals (95% CIs). All statistical analyses were conducted using SPSS 19 (IBM, Chicago, IL, USA), and differences associated with *P* < 0.05 were considered significant.

## Results

Demographic characteristics of MSA, MSA-P, and MSA-C patients with and without FOG are shown in Table 1. Among the 202 patients ultimately enrolled in our study (76 female, 126 male), whose mean age was 64 years, the mean age at MSA onset was 61.53 years and mean MSA duration was 2 years. One hundred and forty (69.3%) were MSA-P and 62 (30.7%) were MSA-C. The distribution of FOG scores was as follows: 0, 105 patients (52.0%); 1, 57 patients (28.2%); 2, 33 patients (16.3%); and 3, 7 patients (3.5%). None of our patients scored a 4. The frequency of FOG was 48.0, 52.1, and 38.7% in the total MSA, MSA-P, and MSA-C patients, respectively. The mean FOG score was 1.48 ± 0.65 for MSA-P group and 1.50 ± 0.59 for MSA-C group. Patients with or without FOG did not differ significantly in age, sex, age at MSA onset, MSA duration, lifestyle, concurrent diseases or medication.

**TABLE 1:** Demographic characteristics of MSA, MSA-P, and MSA-C patients with and without FOG.

Characteristic	Total	MSA			MSA-P			MSA-C		
		**No FOG**	**FOG**	***P***	**No FOG**	**FOG**	***P***	**No FOG**	**FOG**	***P***
N	202	105	97		67	73		38	24	
Male	126 (62.4)	68 (64.8)	58 (59.8)	0.466^a^	46 (68.7)	42 (57.5)	0.174^a^	22 (57.9)	16 (66.7)	0.490^a^
Age, year	64.00 ± 10.46	63.28 ± 10.68	64.79 ± 10.21	0.304^b^	65.40 ± 10.60	65.53 ± 10.34	0.941^b^	59.53 ± 9.88	62.54 ± 9.67	0.241^b^
Age at MSA onset, year	61.53 ± 10.31	60.90 ± 10.40	62.22 ± 10.23	0.368^b^	63.09 ± 10.36	62.85 ± 10.50	0.892^b^	57.05 ± 9.43	60.29 ± 9.29	0.190^b^
MSA duration, year	2.41 ± 2.00	2.26 ± 1.90	2.58 ± 2.12	0.258^b^	2.27 ± 1.88	2.74 ± 2.24	0.184^b^	2.23 ± 1.97	2.08 ± 1.61	0.756^b^
MSA-P	140 (69.3)	67 (63.8)	73 (75.3)	0.078^a^	–	–	–	–	–	–
Smoking history	61 (30.2)	33 (31.4)	28 (28.9)	0.692^a^	22 (32.8)	17 (23.3)	0.208^a^	11 (28.9)	11 (45.8)	0.176^a^
Drinking history	65 (32.2)	36 (34.3)	29 (29.9)	0.505^a^	25 (37.3)	18 (24.7)	0.105^a^	11 (28.9)	11 (45.8)	0.176^a^
**Medication**										
Levodopa	124 (61.4)	62 (59.0)	62 (63.9)	0.478^a^	53 (79.1)	56 (76.7)	0.733^a^	9 (23.7)	6 (25.0)	0.906^a^
Dopamine agonists	23 (11.4)	11 (10.5)	12 (12.4)	0.672^a^	11 (16.4)	9 (12.3)	0.490^a^	0	3 (12.5)	0.054^a^
Amantadine	14 (6.9)	7 (6.7)	7 (7.2)	0.878^a^	4 (6.0)	6 (8.2)	0.607^a^	3 (7.9)	1 (4.2)	0.564^a^
Benzhexol	7 (3.5)	2 (1.9)	5 (5.2)	0.208^a^	2 (3.0)	4 (5.5)	0.468^a^	0	1 (4.2)	0.387^a^
**Comorbidity**										
Hypertension	46 (22.8)	25 (23.8)	21 (21.6)	0.715^a^	18 (26.9)	17 (23.3)	0.625^a^	7 (18.4)	4 (16.7)	0.861^a^
Diabetes mellitus	23 (11.4)	11 (10.5)	12 (12.4)	0.672^a^	6 (9.0)	9 (12.3)	0.519^a^	5 (13.2)	3 (12.5)	0.940^a^

FOG, freezing of gait; MSA, multiple system atrophy; MSA-P, multiple system atrophy with predominately parkinsonism; MSA-C, multiple system atrophy with predominately cerebellar ataxia.

^a^Chi-squared test.

^b^Student’s *t*-test.

**FIGURE 1:**
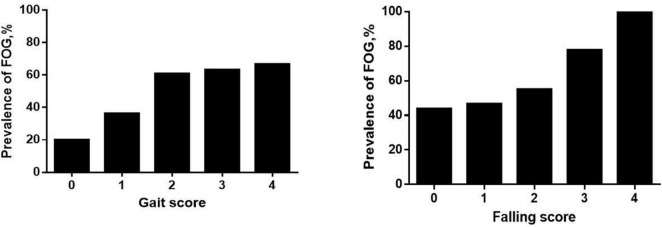
Prevalence of freezing of gait (FOG) among multiple system atrophy (MSA) patients with different UMSARS scores for gait (item 14) and falling (item 8). Higher UMSARS scores indicate worse condition.

**FIGURE 2:**
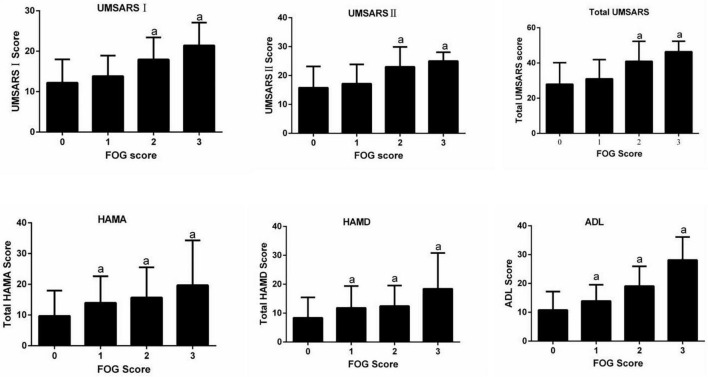
Relationships between FOG severity and clinical variables. ADL, activities of daily living; HAMD, Hamilton Depression Rating Scale; HAMA, Hamilton Anxiety Rating Scale; UMSARS, Unified MSA Rating Scale; UMSARS I, part I (historical review); UMSARS II, part II (motor examination scale); total UMSARS, part I + II. ^a^*p* < 0.05 vs. patients without FOG (Dunnett *post-hoc* test after analysis of variance testing).

In the MSA group, patients with MSA and FOG showed more advanced disease, as reflected on part IV of the UMSARS, as well as greater disability, as reflected by total UMSARS score and subscores on parts I and II (Table 2). They also reported a higher frequency of speech difficulties, falls, gait impairment and balance disorder. They showed significantly higher levels of anxiety and depression symptoms, as well as higher ADL score. Prevalence of FOG showed a tendency to increase with worsening gait and falling (Figure 1). In addition, FOG severity was associated with total UMSARS score and subscores on parts I and II, as well as with scores on the HAMA, HAMD and ADL (Figure 2). Correlation analysis confirmed these relationships (Table 3). In contrast, patients with or without FOG did not differ significantly in other non-motor characteristics such as urinary incontinence, divided attention or cognitive impairment.

**TABLE 2:** Clinical assessments of MSA, MSA-P, and MSA-C patients with and without FOG.

Characteristic	Total	MSA			MSA-P			MSA-C		
		**No FOG**	**FOG**	***P***	**No FOG**	**FOG**	***P***	**No FOG**	**FOG**	***P***
Urinary incontinence	121 (59.9)	60 (57.1)	61 (62.9)	0.405^a^	41 (61.2)	48 (65.8)	0.575^a^	19 (50.0)	13 (54.2)	0.749^a^
Divided attention	58 (28.7)	24 (22.9)	34 (35.1)	0.056^a^	16 (23.9)	27 (37.0)	0.093^a^	8 (21.1)	7 (29.2)	0.467^a^
UMSARS I	13.93 ± 6.03	12.21 ± 5.78	15.79 ± 5.75	<0.001^b^	12.48 ± 5.88	15.77 ± 5.40	0.001^b^	11.74 ± 5.66	15.88 ± 6.84	0.012^b^
Speech difficulties	64 (31.7)	26 (24.8)	38 (39.2)	0.028^a^	10 (14.9)	24 (32.9)	0.013^a^	16 (42.1)	14 (58.3)	0.213^a^
Falls	32 (15.8)	11 (10.5)	21 (21.6)	0.030^a^	4 (6.0)	13 (17.8)	0.032^a^	7 (18.4)	8 (33.3)	0.182^a^
UMSARS II	17.67 ± 7.54	15.78 ± 7.37	19.72 ± 7.21	<0.001^b^	16.45 ± 7.73	20.04 ± 7.07	0.005^b^	14.58 ± 6.62	18.75 ± 7.67	0.027^b^
Gait impairment	101 (50.0)	39 (37.1)	62 (63.9)	<0.001^a^	20 (29.9)	47 (64.4)	<0.001^a^	19 (50.0)	15 (62.5)	0.335^a^
Balance score	2.12 ± 1.67	1.85 ± 1.48	2.42 ± 1.81	0.014^b^	1.48 ± 1.41	2.19 ± 1.64	0.007^b^	2.50 ± 1.39	3.13 ± 2.13	0.167^b^
Total UMSARS score	31.60 ± 12.69	27.98 ± 12.21	35.52 ± 12.08	<0.001^b^	28.93 ± 12.71	35.81 ± 11.61	0.001^b^	26.32 ± 11.24	34.63 ± 13.62	0.011^b^
UMSARS IV	1.85 ± 1.05	1.64 ± 0.95	2.07 ± 1.10	0.003^b^	1.67 ± 1.04	2.04 ± 1.11	0.044^b^	1.58 ± 0.79	2.17 ± 1.09	0.017^b^
HAMA	30.58 ± 25.73	24.96 ± 23.96	36.66 ± 26.31	0.001^b^	25.01 ± 23.87	38.11 ± 26.31	0.003^b^	24.87 ± 24.43	32.25 ± 26.36	0.266^b^
HAMD	10.37 ± 7.75	8.39 ± 7.08	12.52 ± 7.91	<0.001^b^	8.04 ± 6.87	12.44 ± 8.04	0.001^b^	9.00 ± 7.48	12.75 ± 7.66	0.062^b^
MMSE	24.89 ± 4.71	25.50 ± 4.25	24.24 ± 5.09	0.057^b^	25.71 ± 3.81	24.52 ± 5.20	0.052^b^	24.76 ± 4.90	24.29 ± 4.86	0.713^b^
ADL	13.63 ± 7.47	10.79 ± 6.40	16.71 ± 7.36	<0.001^b^	11.57 ± 6.45	17.11 ± 7.60	<0.001^b^	9.42 ± 6.15	15.50 ± 6.57	<0.001^b^
PSQI	7.74 ± 4.71	7.09 ± 4.51	8.44 ± 4.85	0.041^b^	6.76 ± 4.57	8.97 ± 5.08	0.008^b^	7.66 ± 4.40	6.83 ± 3.71	0.449^b^

ADL, activities of daily living; FOG, freezing of gait; MSA, multiple system atrophy; MSA-P, multiple system atrophy with predominately parkinsonism; MSA-C, multiple system atrophy with predominately cerebellar ataxia; HAMD, Hamilton Depression Rating Scale; HAMA, Hamilton Anxiety Rating Scale; MMSE, Mini-Mental State Examination; PSQI, Pittsburgh Sleep Quality Index; UMSARS, Unified MSA Rating Scale; UMSARS I, part I (historical review); UMSARS II, part II (motor examination scale); UMSARS IV, part IV (global disability scale); total UMSARS score, sum of subscores on parts I and II.

^a^Chi-squared test.

^b^Student’s *t*-test.

**TABLE 3:** Relationships of FOG severity with motor and non-motor symptoms.

Symptom or symptom scale	FOG score (severity)	
	**Correlation coefficient**	***P***
UMSARS I	0.388	<0.001
UMSARS II	0.350	<0.001
Total UMSARS	0.403	<0.001
UMSARS IV	0.284	<0.001
HAMA	0.255	<0.001
HAMD	0.292	<0.001
ADL	0.480	<0.001
Falling	0.200	0.004
Gait	0.270	<0.001
Speech	0.155	0.028
Balance	0.152	0.030

ADL, activities of daily living; FOG, freezing of gait; HAMD, Hamilton Depression Rating Scale; HAMA, Hamilton Anxiety Rating Scale; UMSARS, Unified MSA Rating Scale; UMSARS I, part I (historical review); UMSARS II, part II (motor examination scale); UMSARS IV, part IV (global disability scale); total UMSARS, part I + II.

In the MSA-P subgroup, patients with FOG had higher UMSARS-I, UMSARS-II, UMSARS-IV, and total UMSARS scores than those without FOG (Table 2). They also reported a higher frequency of speech difficulties, falls, gait impairment and balance disorder. In addition, they showed significantly higher levels of anxiety, depression and sleep disorders, as well as higher ADL score. In the MSA-C subgroup, patients with FOG showed higher UMSARS-I, UMSARS-II, UMSARS-IV, total UMSARS scores and ADL score than those without FOG (Table 2).

Factors associated with FOG in MSA, MSA-P, and MSA-C patients in the multivariate regression model are shown in Table 4. Higher total UMSARS scores (OR = 1.053, 1.049, and 1.057, respectively) were associated with FOG in MSA, MSA-P, and MSA-C patients, respectively.

**TABLE 4:** Factors associated with FOG in MSA, MSA-P, and MSA-C patients in multivariate regression model.

Variables	MSA			MSA-P			MSA-C		
	**OR**	**95% CI**	***P*-value^a^**	**OR**	**95% CI**	***P*-value^b^**	**OR**	**95% CI**	***P*-value^c^**
Total UMSARS score	1.053	1.027–1.079	<0.001	1.049	1.018–1.081	0.002	1.057	1.010–1.107	0.017

MSA, multiple system atrophy; FOG, freezing of gait; MSA-P, multiple system atrophy with predominately parkinsonism; MSA-C, multiple system atrophy with predominately cerebellar ataxia; UMSARS, unified multiple system atrophy rating scale.

^a^Sex, age, MSA-P subtype, MSA duration, total UMSARS score, and HAMA in the multivariate model.

^b^Sex, age, MSA duration, and total UMSARS score, and HAMA score in the multivariate model.

^c^Sex, age, MSA duration, and total UMSARS score in the multivariate model.

## Discussion

The present study appears to be the largest so far to assess the prevalence, severity and associated clinical features of FOG in MSA. We found that FOG was prevalent in patients with MSA, MSA-P, and MSA-C. We highlighted that greater disease severity was associated with the presence of FOG in MSA as well as in MSA-P and MSA-C patients.

We diagnosed FOG based on UPDRS item 14, which correlates strongly with the recently described FOG questionnaire FOG-Q (Giladi et al., 2009). We found that 48% of our Chinese MSA patients had FOG, similar to the 54% reported for a study of 15 Austrian patients with pathology-confirmed MSA (Muller et al., 2002). However, our prevalence was markedly lower than in two previous reports in China and Israel, which reported 65.9% (Yang et al., 2022) and 75% (Gurevich and Giladi, 2003). We suspect that the high prevalence in the two studies reflects their inclusion of patients had longer disease duration and diagnosis of FOG based on the more specialized FOG-Q. Consistent with the Israeli study, we found that FOG was more prevalent among MSA-P patients than among MSA-C patients, although these differences did not achieve significance in our sample. If these differences are real, they may reflect that MSA-P is associated with brain atrophy more in the basal ganglia than in the cerebellum (Nutt et al., 2011; Wang et al., 2011; Lin et al., 2020). In our sample, FOG prevalence was not associated with age, sex or age at MSA onset, consistent with the Israeli and Austrian studies.

Our findings suggest that there is an association between FOG and the severity of MSA, consistent with the Chinese study (Yang et al., 2022). Our patients with FOG had more severe motor impairment than those without FOG, and higher total UMSARS score was identified as an independent risk factor for FOG. To better understand clinical factors that may be associated with FOG in MSA patients, we explored potential associations between FOG in our sample and clinical parameters previously associated with FOG in PD (Giladi et al., 2001). We found an association between MSA-related speech difficulties and FOG, consistent with a previous analysis of Austrian patients with postmortem-confirmed atypical parkinsonism (Muller et al., 2002). It would be interesting to identify which speech disturbances may relate specifically to FOG. Moreover, we found that the frequency of freezing episodes in patients with FOG increased proportionally with the severity of falling and gait impairment (Figure 1), which is in line with the “threshold model” in PD (Plotnik et al., 2012). This model stipulates that FOG occurs when motor deficits accumulate to the point of motor breakdown, which may explain why patients with FOG show worse stride amplitude, gait coordination and variability in step timing than patients without FOG. This model may also explain why our MSA patients with FOG were at higher risk of falling than those without FOG. Nevertheless, it is unclear whether balance impairment is an accompanying symptom of FOG or a contributor to it, so future studies should examine this question more closely.

Freezing of gait in PD patients has been linked to various non-motor symptoms, such as urinary incontinence, divided attention, sleep disorder, cognitive impairment, anxiety and depression (Ou et al., 2018; Gao et al., 2020). In contrast, FOG in our MSA patients was not associated with divided attention. Similarly, we found no association of FOG with urinary incontinence or cognitive function, which is inconsistent with a report on US patients with parkinsonian syndromes (Giladi et al., 1997). In addition, many studies in PD have linked FOG to worse cognition across several domains, especially in executive function (Kim et al., 2018; Gao et al., 2020). Some of this discrepancy may reflect how studies have assessed cognitive function: the MMSE, used in the present study, may not be as precise as other cognitive scales such as the Montreal Cognitive Assessment or Addenbrooke’s Cognitive Examination for detecting frontal lobe dysfunction. Future studies should explore this question.

Our study provides the evidence linking both anxiety and depression to FOG in MSA. This would be consistent with the “interference model” in PD (Lewis and Barker, 2009), which suggests that interconnections between the limbic and motor circuits within the basal ganglia allow emotional input to interfere with motor output, thus resulting in FOG. Furthermore, we found that patients with FOG showed worse ADL than whose without it, which probably reflects that it reduces patients’ independence and mobility. The implication is that FOG reduces the quality of life of MSA patients, which should be explored in detail in future work.

Our data provide some hints that pharmacological factors may influence risk of FOG in MSA. We did not detect associations of FOG with use of levodopa, dopamine agonists or amantadine. Unfortunately, the dose of drugs taken for MSA patients at the time of the visit were not recorded. So, we can’t calculate the levodopa daily equivalent dose. We found that use of benzhexol was more prevalent among our patients with FOG than among those without it, although this difference did not achieve significance. Consistent with our results, a study of French patients with PD found an association between FOG and use of anti-muscarinics (Perez-Lloret et al., 2014). These observations, together with the well-established fact that gait and posture involve loss of cholinergic neurons in the pedunculopontine nucleus (Devos et al., 2010; Craig et al., 2020), argue that MSA patients with FOG should avoid anti-muscarinics.

Our findings should be interpreted with caution in light of several limitations. First, we did not screen for spinocerebellar ataxia (SCA) genes to exclude the common forms of SCA before a MSA diagnosis. Second, although we examined a reasonably large number of patients, all came from two centers, which raises the risk of selection bias and potentially limits the generalizability of our results. Our results should be confirmed in larger, preferably longitudinal studies. Third, we considered FOG as a single syndrome, without dividing it into subtypes involving, for example, hesitation upon starting, hesitation upon turning, or freezing while walking. Fourth, we diagnosed FOG based on the subjective, self-report measure of UPDRS item 14. This renders our diagnosis vulnerable to recall bias and confounding with other gait disturbances such as primary progressive freezing of gait. It would be more accurate to diagnose FOG based on videos of patients’ walking (Lord et al., 2020).

## Conclusion

The present study demonstrated that freezing of gait was common in Chinese patients with MSA, MSA-P, and MSA-C. Greater disease severity was associated with freezing of gait in patients with MSA, MSA-P, and MSA-C. Therefore, more attention should be paid to FOG in patients with MSA. Our findings also may have implications for rehabilitation and treatment of MSA patients with FOG.

## Data availability statement

The original contributions presented in this study are included in the article/supplementary material, further inquiries can be directed to the corresponding authors.

## Ethics statement

The studies involving human participants were reviewed and approved by the Ethics Committee of West China Hospital of Sichuan University. The patients/participants provided their written informed consent to participate in this study.

## Author contributions

YC collected the data, conducted the statistical analysis, interpreted the data, and wrote the manuscript. HW and HH collected the data. YX and YMC revised the manuscript. All authors contributed to the article and approved the submitted version.
